# Supplementary material for: Population-based tract-to-region connectome of the human brain and its hierarchical topology
Source: Nat Commun. 2022 Aug 22;13:4933. doi: 10.1038/s41467-022-32595-4 (PMC9395399; doi:10.1038/s41467-022-32595-4)
Supplement: Supplementary file 1 — Supplementary Information [file 41467_2022_32595_MOESM1_ESM.pdf]

# Supplementary Information

## Supplementary Table 1

| Abbreviation | Full Name | Other Nomenclatures |
|---|---|---|
| AF | Arcuate Fasciculus | |
| C_FPH | Cingulum, Frontal Parahippocampal Segment | |
| C_FP | Cingulum, Frontal Parietal Segment | |
| C_PH | Cingulum, Parahippocampal Segment | |
| C_PHP | Cingulum, Parahippocampal Parietal Segment | Sledge Runner Fasciculus |
| C_PR | Cingulum, Parolfactory Segment | |
| CBT | Corticobulbar Tract | |
| CST | Corticospinal Tract | |
| CStr_A | Corticostriatal Tract, Anterior Segment | |
| CStr_P | Corticostriatal Tract, Posterior Segment | |
| CStr_S | Corticostriatal Tract, Superior Segment | |
| CTh_A | Coricothalamic Tract, Anterior Segment | Anterior Thalamic Radiation |
| CTh_P | Coricothalamic Tract, Posterior Segment | Posterior Thalamic Radiation |
| CTh_S | Coricothalamic Tract, Superior Segment | Superior Thalamic Radiation |
| F | Fornix | |
| FAT | Frontal Aslant Tract | |
| IFOF | Inferior Fronto Occipital Fasciculus | |
| ILF | Inferior Longitudinal Fasciculus | |
| MdLF | Middle Longitudinal Fasciculus | |
| OR | Optic Radiation | Meyer's Loop |
| TPAT | Temporo-Parietal Aslant Tract | Posterior AF, Vertical AF, SLF-tp |
| SLF_I | Superior Longitudinal Fasciculus 1 | |
| SLF_II | Superior Longitudinal Fasciculus 2 | Dorsal SLF |
| SLF_III | Superior Longitudinal Fasciculus 3 | Ventral SLF, Long segment of AF |
| UF | Uncinate Fasciculus | |
| VOF | Vertical Occipital Fasciculus | |

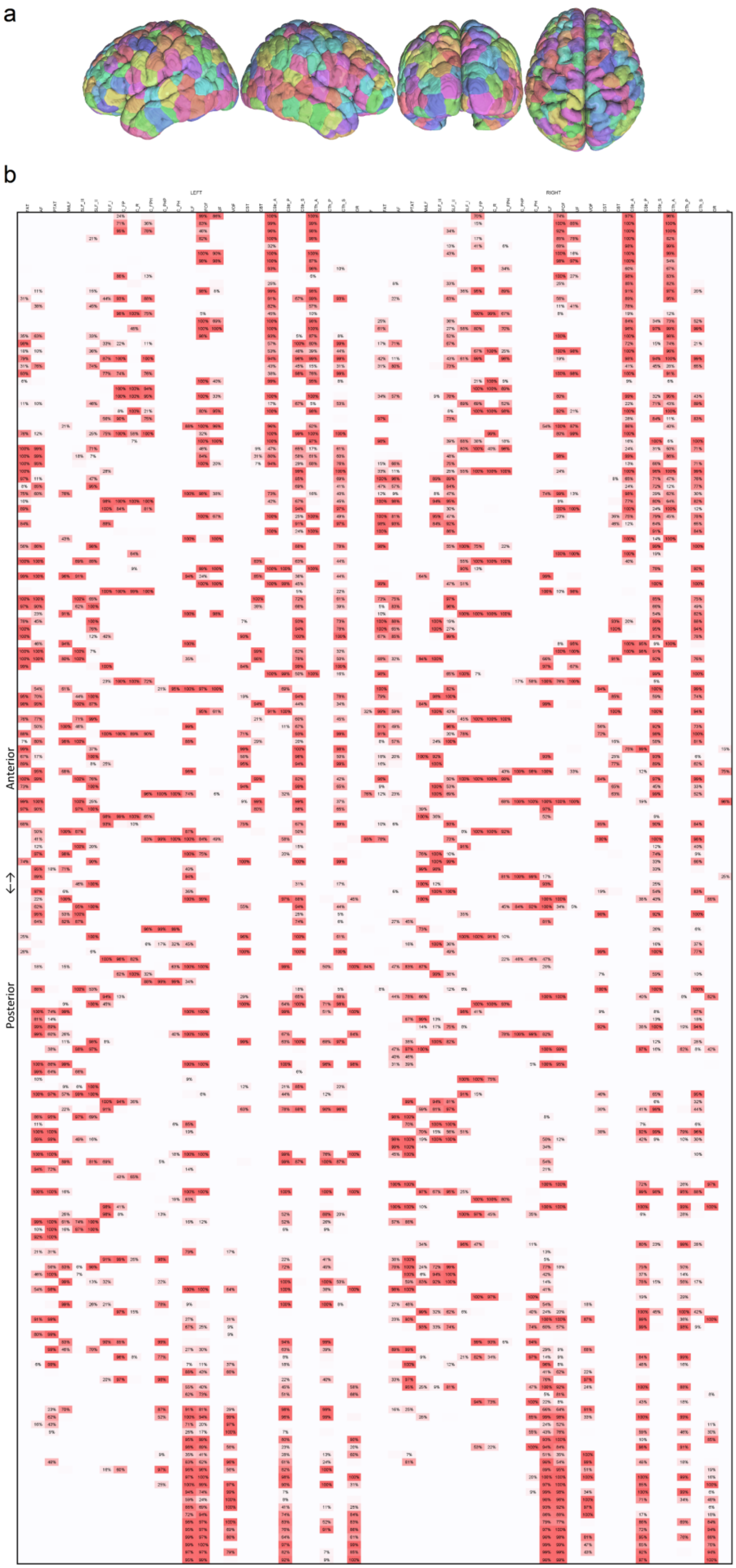
a
b
LEFT
RIGHT
Anterior
Posterior

**Supplementary Fig. 1: Tract-to-region connectome using a random parcellation.**

**(a)** The random parcellation was derived from level 33 of Craddock's fine-grained random parcellations18 after wrapping it to the ICBM152 space. **(b)** The tract-to-region connectome based on Craddock's parcellation also showed 87.09% of the matrix entries (8152 out of 9360) had probability values ranging between 95% and 5%, similar to results based on other anatomy-based parcellation.

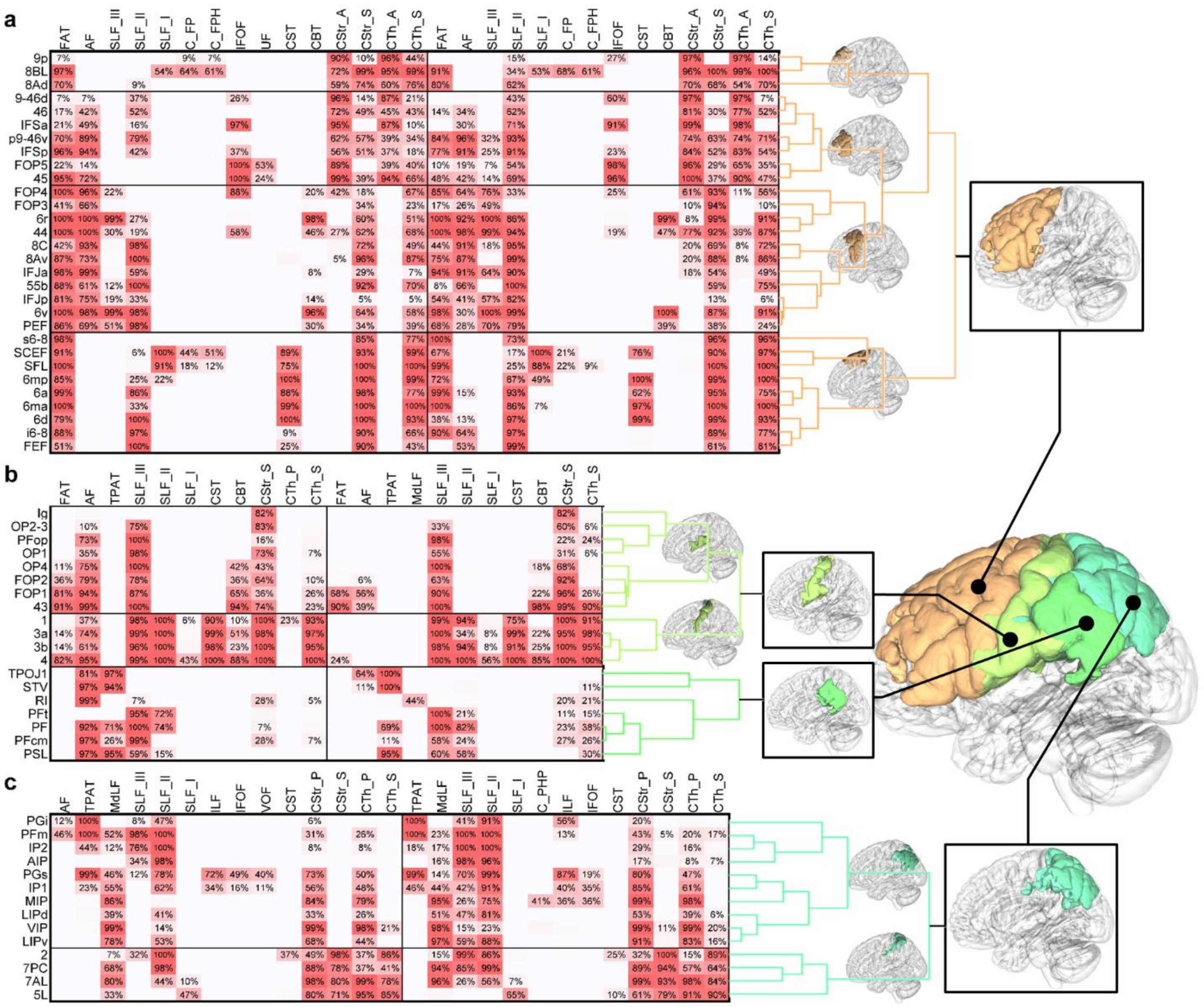


**Supplementary Fig. 2: Dorsal network and its subnetworks**

The figure shows the tract-to-region connections of the dorsal networks. The rows are cortical regions, whereas the columns are the white matter tracts. The left half of the matrix corresponds to the left hemisphere, whereas the right corresponds to the right hemisphere. **(a)** The first part of the dorsal network includes most of the frontal lobe regions connected by FAT, SLF II, and AF. The anterior border is right behind the prefrontal lobe, whereas the posterior border is located on the precentral gyrus right before the primary motor area (area 4). **(b)** The second part of the dorsal network is located at the anterior parietal lobe and slightly extended into the precentral gyrus. Anteriorly, it includes part of the precentral gyrus (area 4), postcentral gyrus (area 1, 3a, and 3b), and the operculum. Posteriorly, it includes the supramarginal and angular gyrus and partly extends to the supratemporal gyrus (e.g., TPOJ1 and STV). These regions are connected by SLF III. **(c)** The third part of the dorsal network is located at the superior parietal lobe and connected by MdLF. The anterior border is located at area 2 of the primary somatosensory cortex, whereas the posterior border ends at area PGs. MATLAB (MathWorks©, https://www.mathworks.com/ ) was used to create the tree diagrams.

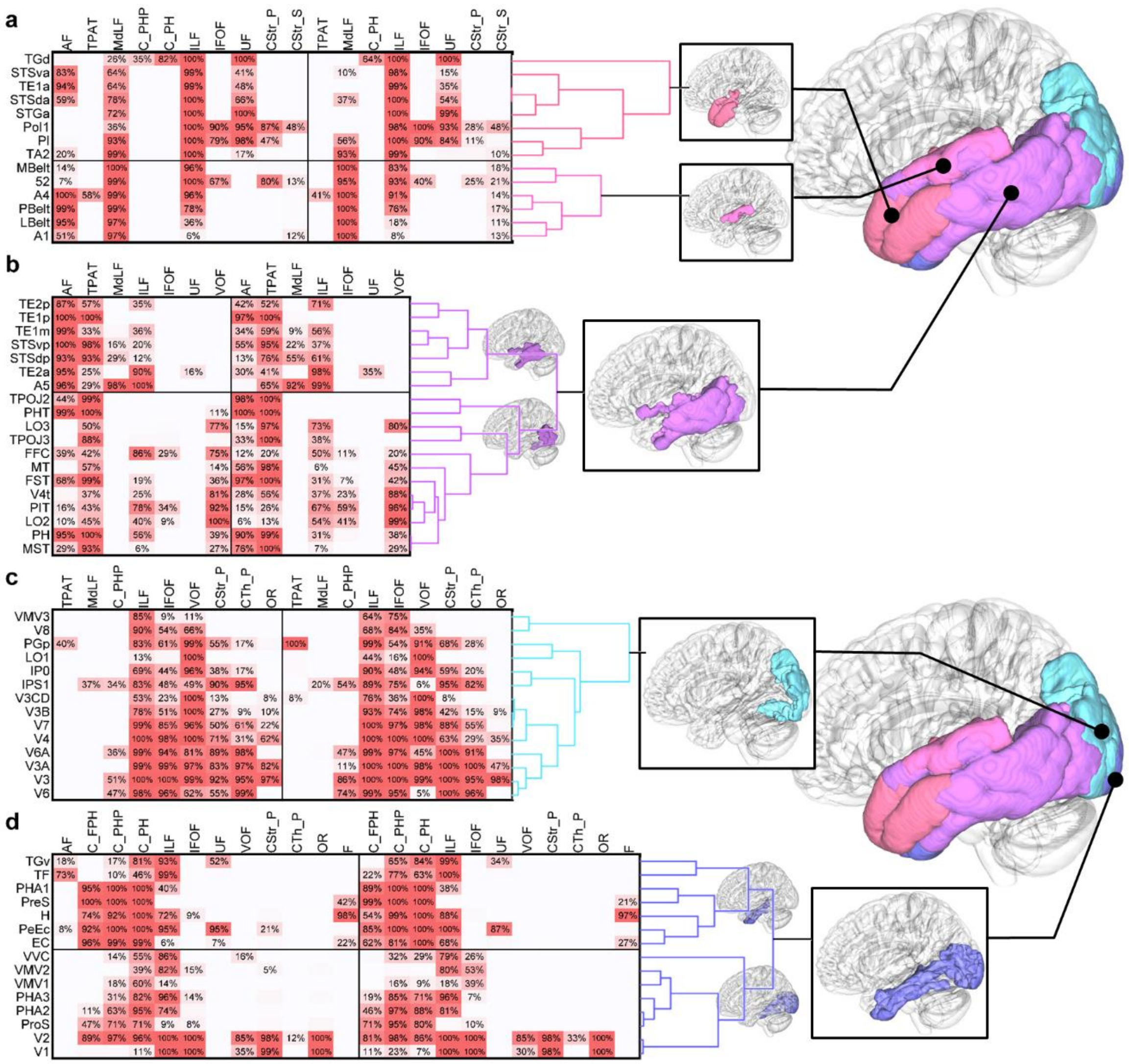


**Supplementary Fig. 3: Ventral network and its subnetworks**

The figure shows the tract-to-region connections of the ventral networks. The rows are cortical regions, whereas the columns are the white matter tracts. The left half of the matrix corresponds to the left hemisphere, whereas the right corresponds to the right hemisphere. **(a)** The first part of the ventral network is located at the anterior temporal pole and superior temporal gyrus. These regions are connected with MdLF and ILF. **(b)** The second part includes most of the middle temporal and inferior temporal regions connected with AF, TPAP, and ILF. **(c)** The third part includes the lateral part of the occipital lobe and encompasses most of the visual cortex (V3-V8), except for V1 and V2. These regions are connected by ILF, IFOF, and VOF. **(d)** The fourth part includes the medial part of the occipital lobe, including V1 and V2, in addition to the medial temporal regions around the parahippocampal regions. The regions are connected by pathways from ILF and cingulum pathways such as C_FPH, C_PHP, and C_PH. C_PHP is also known as the sledge runner fasciculus (Koutsarnakis et al., 2019; Vergani et al., 2014), which connects the medial occipital lobe and parahippocampal gyrus through the isthmus of the cingulum. Overall, the ventral network includes the conventional definition of the temporal and

occipital lobe. MATLAB (MathWorks©, https://www.mathworks.com/ ) was used to create the tree diagrams.

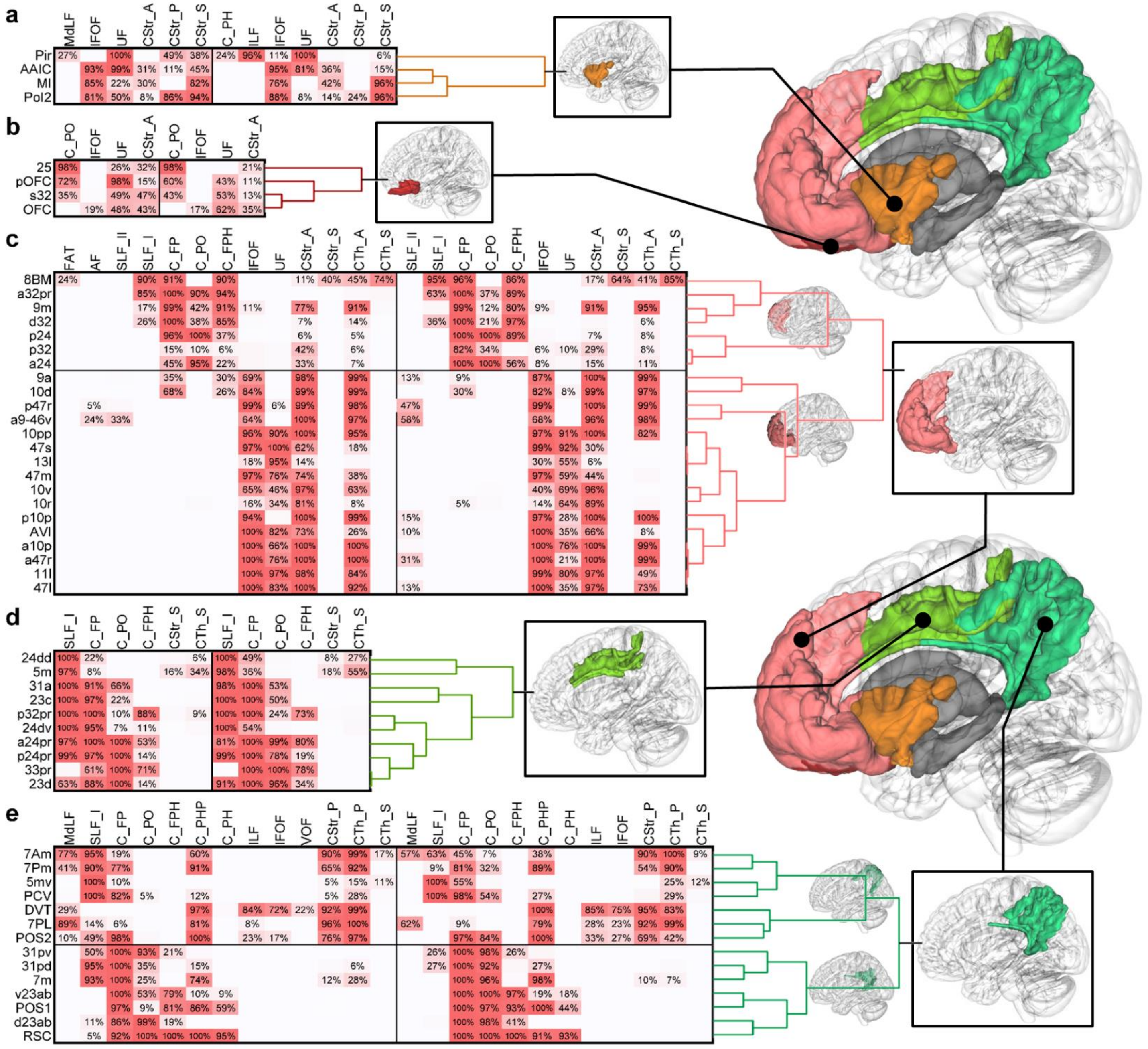

**Supplementary Fig. 4: Limbic network and its subnetworks**

The figure shows the tract-to-region connections of the limbic networks. The rows are cortical regions, whereas the columns are the white matter tracts. The left half of the matrix corresponds to the left hemisphere, whereas the right corresponds to the right hemisphere. The limbic network is characterized by connections with the cingulum. The network can be further divided into subnetworks including **(a)** the insula, **(b)** the olfactory cortex, **(c)** the prefrontal cortex, **(d)** the superior medial frontal cingulum gyrus, and **(e)** The parietal cingulum. The limbic network has close relations with the hippocampus and basal ganglions (marked as dark gray regions), which are not included in the HCP-

MMP parcellation and are thus not included in the clustering analysis. Overall, the limbic network servers are the bridging structure between the dorsal and ventral networks. MATLAB (MathWorks©, https://www.mathworks.com/ ) was used to create the tree diagrams.

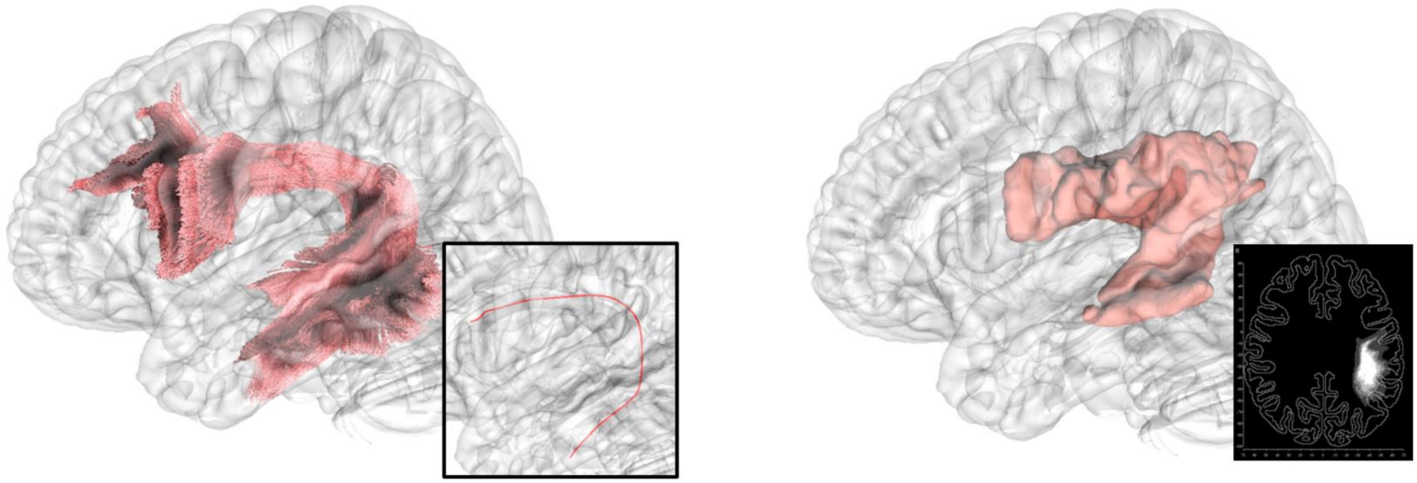

**Supplementary Fig. 5: Track-based versus voxel-based atlas of the left arcuate fasciculus**

The track-based atlases provide the connection trajectories of white matter pathways, whereas the voxel-based atlases are voxel-wise masks with no trajectory information.
